# Risk–benefit balance of blood cultures among patients with stage IV cancer in unplanned admission: a nationwide propensity score–weighted study in Japan

**DOI:** 10.1093/jac/dkaf368

**Published:** 2025-10-03

**Authors:** Yuki Hashimoto, Norihiko Inoue, Takuaki Tani, Shinobu Imai

**Affiliations:** Department of Clinical Data Management and Research, Clinical Research Center, National Hospital Organization Headquarters, 2-5-21 Higashigaoka, Meguroku, Tokyo 152-8621, Japan; Department of Clinical Data Management and Research, Clinical Research Center, National Hospital Organization Headquarters, 2-5-21 Higashigaoka, Meguroku, Tokyo 152-8621, Japan; Department of Pharmacoepidemiology, Showa University Graduate School of Pharmacy, Tokyo, Japan; Department of Pharmacoepidemiology, Showa University Graduate School of Pharmacy, Tokyo, Japan

## Abstract

**Objectives:**

Infection commonly causes unplanned admission in patients with stage IV cancer; however, the risk–benefit balance of blood cultures remains unclear. We evaluated clinical outcomes of blood culture among patients with stage IV cancer in unplanned admission.

**Methods:**

We conducted a retrospective cohort study across Japan (April 2016 to March 2023). Patients with stage IV solid cancer receiving IV antimicrobials in unplanned admission were divided into blood culture (BC) and no blood culture (NC) groups. After overlap propensity score weighting, we compared mortality, functional disability, length of stay from antimicrobial initiation to discharge (LOS), and total hospitalization costs. Mortality risk was assessed using modified Poisson regression. Interaction tests were used to evaluate subgroup differences. Composite outcomes (mortality and functional disability) were assessed using a win-ratio approach (a hierarchical comparison of outcomes, prioritizing mortality over functional status).

**Results:**

Among 10 915 patients (BC: 4029, NC: 6886), mortality was lower in the BC than the NC group (23.9% versus 29.2%; risk ratio: 0.81; 95% CI, 0.75–0.88). Mortality reduction was significantly greater in patients with prior chemotherapy or immunosuppressive agents. Composite outcomes were more favourable in the BC than the NC group (win ratio: 1.22; 95% CI, 1.13–1.32). However, BCs were associated with longer LOS (1.0 days; 95% CI, 0.0–1.9) and higher hospitalization costs (345.0 USD; 95% CI, 72.5–628.1).

**Conclusions:**

BCs were associated with reduced mortality in patients with stage IV cancer, particularly those with immunosuppression. These findings may support personalized decision-making and resource allocation.

## Introduction

Infection is a leading cause of unplanned admission among patients with cancer^1^ owing to their compromised immune system, malnutrition and the presence of invasive medical devices.^2,3^ The majority of patients with advanced cancer receive antimicrobials for infections, reaching up to 90% in the last week of life.^2,4^

Blood cultures are essential for identifying causative bacteria and their antimicrobial resistance patterns, guiding the choice of antimicrobial therapy.^5^ Although blood cultures are recommended for immunocompromised patients in emergency departments based on expert opinion,^6^ limited evidence exists for reduction in unnecessary blood cultures among adult patients, especially immunocompromised hosts.^7^ For patients with stage IV cancer in unplanned admission, including those in the terminal stage, antimicrobial therapy might not alter the clinical outcome.^8^ Multiple blood sampling can lead to discomfort, phlebitis, psychological burden and inefficient use of medical resources.^9,10^ Unnecessary testing and treatment based on false-positive blood culture results owing to contamination can increase medical costs by approximately 50% and the length of stay by 64%.^9^

To balance between benefits and risks requires identifying patients for whom blood cultures would improve life expectancy and quality of life. In surviving patients, functional status at discharge is equally crucial because it directly affects the care burden on families and society. Previous studies of general cancer have focused on in-hospital mortality without functional status,^11^ whereas those of terminal cancer included only patients who died during hospitalization,^10,12^ overlooking the functional outcomes for surviving patients.

This study aimed to evaluate the clinical outcomes among patients with stage IV cancer receiving antimicrobial therapy in unplanned admission who did or did not receive blood cultures before antimicrobial initiation. Our findings could provide empirical evidence for clinical decision-making based on the risk–benefit balance.

## Methods

### Study design and data source

In this retrospective cohort study, we assessed whether blood cultures could reduce mortality and improve functional status in patients with stage IV cancer receiving antimicrobial therapy in unplanned admission. Data were collected from 140 National Hospital Organization (NHO) hospitals in Japan.^13^ The NHO maintains two databases: an administrative claims database and a clinical information database.^14^ The administrative claims database contains comprehensive information on patient demographics, health condition, medical costs, diagnosis, procedures and medications. The clinical information database contains daily records of medical charts, vital signs, and laboratory and microbiology test results. This study was conducted in accordance with the Declaration of Helsinki and was approved by the Institutional Review Board of Showa University (2023-129-A). Individual informed consent was waived due to the retrospective design, with an opt-out opportunity provided via the hospital website. Results were reported according to the Strengthening the Reporting of Observational Studies in Epidemiology guidelines.^15^

### Participants

This study included adult patients (aged ≥18 years) with stage IV solid cancer admitted to NHO hospitals from April 2016 to March 2023 who started IV antimicrobials within 1 day after unplanned admission. These criteria targeted patients with stage IV solid cancer who initiated antimicrobials for suspected infection, as blood cultures are recommended for immunocompromised patients.^6^

We excluded patients who underwent surgery under general anaesthesia on or before the day of antimicrobial initiation,^16^ were diagnosed with coronavirus disease 2019 (COVID-19) during hospitalization, had HIV infection,^16^ were diagnosed with febrile neutropenia (FN) at admission, were pregnant^16^ or had missing Barthel Index (BI) data at discharge. These exclusion criteria were applied because surgically treated patients might receive antimicrobials for prophylaxis infection; patients with COVID-19 or HIV infection and pregnancy may require nonstandard antimicrobial use; patients with FN require blood cultures as standard of care,^17^ leading to a lack of patients with FN in the no blood culture group for comparison; and patients missing BI data could not be evaluated for the outcome. FN was categorized using the ICD-10 as D70.^18^

Patients were divided into blood culture (BC) and no blood culture (NC) groups based on whether blood cultures were collected within 2 days preceding or on the same day as antimicrobial initiation.^5,19^

### Outcome variables

Primary outcomes were in-hospital mortality and functional disability at discharge. Functional status was assessed at discharge in survivors only. Functional disability was categorized as bedridden (discharge BI ≤35)^20^ or severe dependence (discharge BI 40–60).^21^ To ensure these outcomes captured only patients with a worsened or persistently poor functional status, this categorization was applied only if a patient's discharge BI did not exceed their admission BI. The BI evaluated the functional status on a scale of 0–100 in 5-point increments, with lower scores indicating greater physical dependence.^21^

Secondary outcomes were length of stay from antimicrobial initiation to discharge (LOS), days of therapy (DOTs) of IV antimicrobials per 100 LOS-days, DOTs of broad-spectrum IV antimicrobials^22^ per 100 LOS-days, and healthcare costs. DOTs were used to measure the appropriate antimicrobial use,^23^ determining whether blood cultures were associated with reduction in antimicrobial use. Broad-spectrum antimicrobials included aminoglycosides, antipseudomonal penicillin, carbapenem, fluoroquinolone, fourth-generation cephalosporins, glycopeptides, lipopeptides and oxazolidinones. Healthcare costs were categorized as total, medical consultation, medication, surgical procedure, laboratory tests, hospital stay and others. Costs were converted from Japanese yen (JPY) into US dollars (USD) at 150 JPY to 1 USD.

### Other variables

We collected the following covariates before antimicrobial initiation: age; sex; BMI; functional status [bedridden (BI ≤ 35), severe dependence (BI 40–60), and mild or no dependence (BI ≥ 60)]; primary cancer site (breast, colorectal, liver, lung, pancreas, stomach and others); infectious site diagnosed at admission (blood, genitourinary, intra-abdominal, pulmonary, and others); initial use of broad-spectrum antimicrobials;^22^ Charlson Comorbidity Index (CCI);^18^ medical history within 4 weeks prior to antimicrobial initiation (chemotherapy, radiation, surgery and immunosuppressive agents); admission after January 2020 (after COVID-19 pandemic); ICU admission; palliative care intervention;^4^ oxygen inhalation; mechanical ventilation; vasopressor use; body temperature (BT) <36°C or >38℃;^24^ Glasgow Coma Scale (GCS) <15^24^ based on the Japan Coma Scale;^25^ heart rate (HR) >90 beats/min;^24^ systolic blood pressure (sBP) ≤100 mmHg;^24^ laboratory test values [albumin,^26^ absolute neutrophil count (ANC) <1000 cells/µL,^4^ C-reactive protein (CRP),^27^ creatinine,^24^ haemoglobin, platelet count (Plt),^24^ total bilirubin (T-Bil),^24^ and WBC count >12 000 cells/µL^24^]; and hospital characteristics (<300, 300–499, or ≥500 beds and rural hospital status classified as the depopulation group based on the population density).^28^

Age, sex, BMI, functional status, primary cancer site, CCI, GCS and hospital characteristics were assessed at admission. ICU admission, palliative care intervention, oxygen inhalation, mechanical ventilation, vasopressor use, BT, HR, sBP and laboratory test values were assessed within 2 days before antimicrobial initiation. The primary cancer site, infection site, and CCI were categorized using the ICD-10^18^ (Table S1, available as Supplementary data at *JAC* Online).

In the BC group, we investigated blood culture positivity rate, types of bacteria and their antimicrobial susceptibility. The types of bacteria and antimicrobial susceptibility were determined using the Japan Nosocomial Infections Surveillance System definitions.^29^ To evaluate the effect of blood culture results on antimicrobial stewardship, we compared DOTs for all IV antimicrobials and broad-spectrum IV antimicrobials per 100 LOS-days between patients with positive and negative blood culture results. Among those with positive cultures, we further compared DOTs between patients with susceptible and resistant isolates. An isolate was defined as ‘resistant’ if antimicrobial susceptibility testing reported resistance to at least one antimicrobial agent; otherwise, it was defined as ‘susceptible’.

### Statistical analysis

We used overlap propensity-score weighting to adjust for potential confounders between the BC and NC groups.^30^ This method is robust for varying treatment probabilities because it focuses the analysis on the population with clinical equipoise, which increases the precision and stability of the effect estimate. The propensity score was calculated using a logistic regression model that included all variables as covariates. We evaluated the balance in potential confounding variables between groups before and after weighting using the standardized mean difference (SMD). An SMD value ≤0.1 was considered well balanced.^31^ Missing values were imputed using ‘missRanger’, a random forest-based algorithm,^32^ which assumes that the data are missing at random.

We compared clinical outcomes between groups after weighting. Continuous variables were reported as mean and SD or IQR, depending on the distribution, and compared between groups using the Wilcoxon rank-sum test or the Welch test. Categorical variables were expressed as proportions and compared between groups using the chi-square test.

The risk ratio (RR) with 95% CI for in-hospital mortality was estimated using modified Poisson regression.^33^ The composite outcomes of in-hospital mortality and functional disability were compared between groups using a win-ratio approach,^34^ allowing hierarchical comparison of multiple outcomes, with a ratio >1 indicating a good result (Figure S1). For this analysis, these outcomes were prioritized in the following order: (i) in-hospital mortality, (ii) becoming bedridden, and (iii) becoming severely dependent. Each patient in the BC group was compared with each patient in the NC group using a non-parametric generalized pairwise comparison following the hierarchy of mortality, bedridden and severe dependence. A win was recorded for the BC group if their patient had a better outcome than the NC group. Owing to the possibility of multiple ties, we also calculated the win odds. The differences in LOS and total healthcare costs between groups were calculated as secondary outcomes, assuming a gamma distribution.^35^

To assess the potential heterogeneity of the effect of blood cultures, we conducted subgroup analyses by patient characteristics: age (18–64, 65–74, 75–84, or ≥85 years), sex, BMI (<18.5, 18.5–24.9, or ≥25.0 kg/m^2^), functional status (bedridden or not), CCI (0 or ≥1), and medication (initial use of broad-spectrum antimicrobials, and history of chemotherapy or immunosuppressive agents). The heterogeneity of the treatment effect across these subgroups was tested by introducing an interaction term into the modified Poisson regression models for the primary outcome of in-hospital mortality.

Sensitivity analyses were conducted to test the robustness of our findings. These included an analysis restricted to patients admitted after the COVID-19 pandemic (the pandemic effect); a complete case analysis (the missing-value imputation effect); an analysis of patients receiving IV antimicrobials for three consecutive days (the short-term antimicrobial use effect); an analysis excluding patients who underwent blood culture after IV antimicrobial initiation from the NC group (the blood culture effect after antimicrobial initiation); and an analysis of patients with LOS ≤28 days (the prolonged hospitalization effect). Additionally, we performed a specific sensitivity analysis for in-hospital mortality, which included patients with missing BI data at discharge.

All statistical analyses were performed using R version 4.3.1 (R Foundation for Statistical Computing, Vienna, Austria). Two-sided *P* < 0.05 was considered statistically significant.

## Results

**Figure 1.**
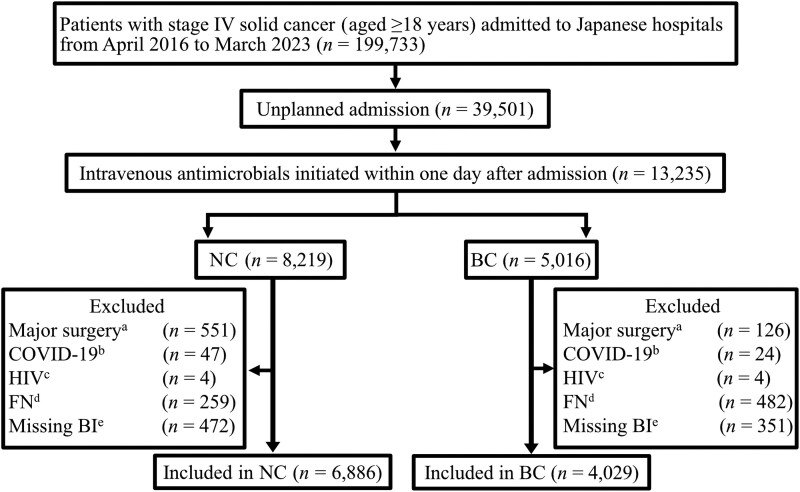
Flow diagram of the enrolment of study participants. ^a^Patients underwent surgery under general anaesthesia on or before the day of antimicrobial initiation after admission. ^b^Patients were diagnosed with COVID-19 during hospitalization. ^c^Patients had HIV infection. ^d^Patients were diagnosed with febrile neutropenia at admission. ^e^Patients with missing Barthel Index at discharge. BC, blood culture group; COVID-19, coronavirus disease 2019; FN, febrile neutropenia; NC, no blood culture group.

In total, 10 915 patients (6886 in the NC group and 4029 in the BC group) from 63 hospitals were included (Figure 1), with no pregnant patients in either group. We imputed missing data for 13 covariates (maximum missing rate <20%; Table S2).

In the BC group, 16.5% of patients had positive blood cultures, and CoNS were detected in 2.1% of patients (Table S3). Additionally, 10.9% of patients had antimicrobial-resistant bacteria. Patients with negative blood culture had shorter DOTs per 100-LOS for all IV antimicrobials than patients with positive blood culture [61.4 (95% CI, 60.4–62.4) versus 69.5 (95% CI, 67.3–71.8); *P* < 0.001], and DOTs per 100-LOS for broad-spectrum IV antimicrobials were similar between these patients (Table S4). However, patients with antimicrobial-susceptible bacteria had DOTs for all IV antimicrobials similar to those of patients with antimicrobial-resistant bacteria, but had shorter DOTs for broad-spectrum IV antimicrobials than those of patients with antimicrobial-resistant bacteria [27.2 (95% CI, 23.1–31.4) versus 32.8 (95% CI, 29.6–36.1); *P* = 0.04] (Table S5).

**Figure 2.**
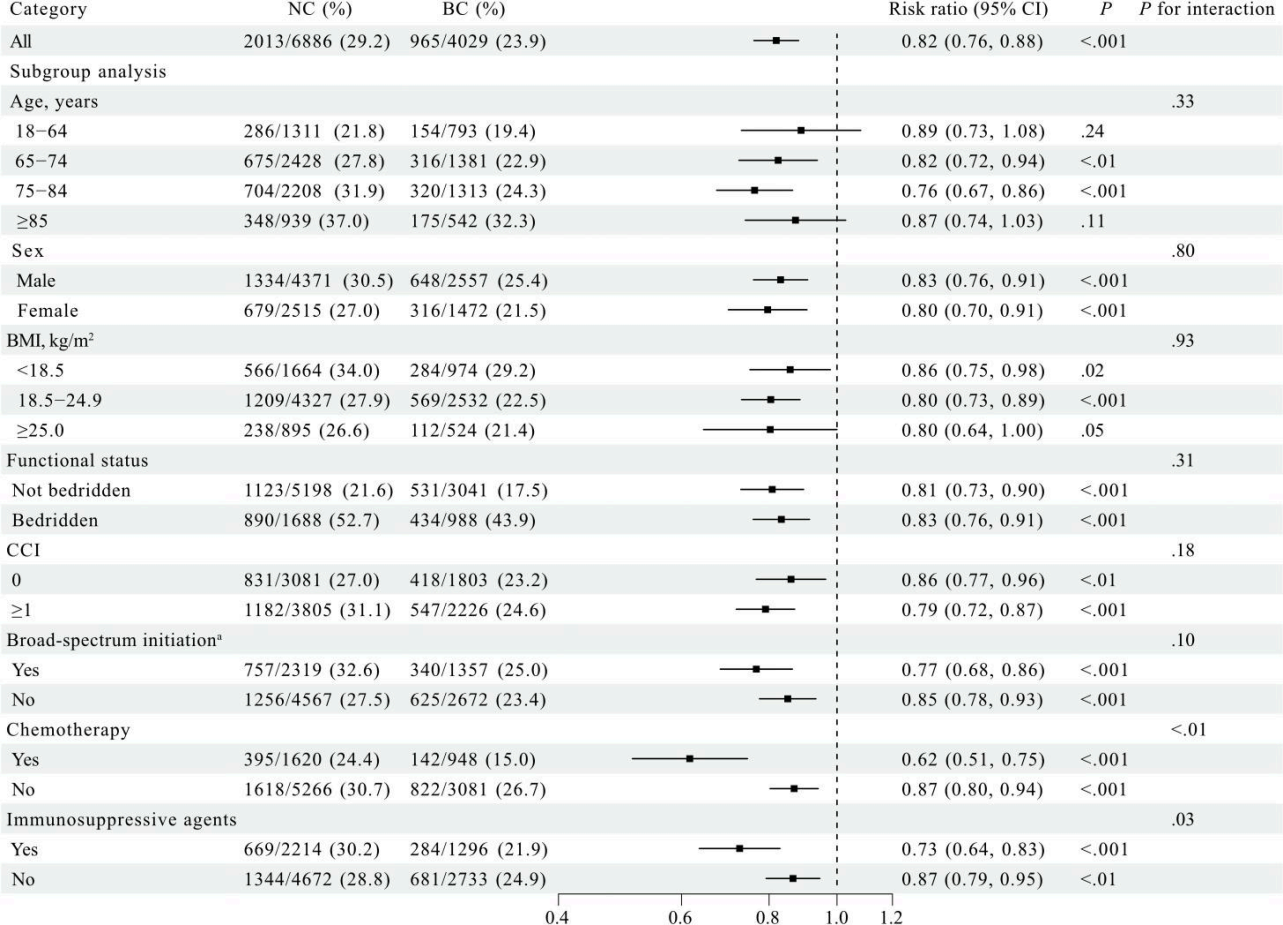
Forest plot showing the risk ratios by subgroup analyses after overlap weighting. ^a^Patients were initially started on broad-spectrum antibiotics. BC, blood culture group; CCI, Charlson Comorbidity Index; COVID-19, coronavirus disease 2019; NC, no blood culture group.

After weighting, all baseline characteristics were well balanced between groups (Table 1). The in-hospital mortality was 23.9% (965/4029) in the BC group and 29.2% (2013/6886) in the NC group. Using the weighted RR (Figure 2), the BC group had a significantly lower mortality rate than the NC group (RR: 0.82; 95% CI, 0.76–0.88; *P* < 0.001). In subgroup analysis, mortality reduction associated with BC was significantly greater among patients with prior chemotherapy (RR: 0.62; 95% CI, 0.51–0.75; *P* for interaction <0.01) or immunosuppressive agents (RR: 0.73; 95% CI, 0.64–0.83; *P* for interaction = 0.03). Although the interaction test for age was not significant (*P* for interaction = 0.33), the 95% CI for RR included the null value of 1 in patients aged 18–64 (0.89; 95% CI, 0.73–1.08) and ≥85 years (0.87; 95% CI, 0.74–1.03). Sensitivity analyses showed similar results to the overall analysis (Figure S2).

**Table 1.** Characteristics of patients with stage IV cancer before and after overlap weighting according to blood culture status

Variable	Before weighting			After weighting		
	NC *n* = 6886	BC *n* = 4029	SMD	NC *n* = 6886	BC *n* = 4029	SMD
Age, mean (SD), y	72.9 (10.9)	72.3 (11.2)	0.05	72.7 (10.9)	72.7 (11.1)	0.00
Sex, *n* (%)			0.01			0.00
Male	4364 (63.4)	2543 (63.1)		4371 (63.5)	2557 (63.5)	
Female	2522 (36.6)	1486 (36.9)		2515 (36.5)	1472 (36.5)	
BMI, *n* (%), kg/m^2^			0.01			0.00
<18.5	1644 (23.9)	953 (23.7)		1664 (24.2)	974 (24.2)	
18.5–24.9	4295 (62.4)	2533 (62.9)		4327 (62.8)	2532 (62.8)	
≥25.0	947 (13.8)	543 (13.5)		895 (13.0)	524 (13.0)	
Functional status, *n* (%)			0.10			0.00
Mild or no dependence	4452 (64.7)	2449 (60.8)		4261 (61.9)	2493 (61.9)	
Severe dependence	909 (13.2)	524 (13.0)		938 (13.6)	549 (13.6)	
Bedridden	1525 (22.1)	1056 (26.2)		1688 (24.5)	988 (24.5)	
Primary cancer site, *n* (%)			0.09			0.00
Breast	90 (1.3)	79 (2.0)		114 (1.7)	67 (1.7)	
Colorectal	1357 (19.7)	699 (17.3)		1230 (17.9)	720 (17.9)	
Liver	259 (3.8)	177 (4.4)		279 (4.0)	163 (4.0)	
Lung	1681 (24.4)	969 (24.1)		1752 (25.4)	1025 (25.4)	
Pancreas	1093 (15.9)	625 (15.5)		1057 (15.4)	619 (15.4)	
Stomach	465 (6.8)	323 (8.0)		514 (7.5)	301 (7.5)	
Others	1941 (28.2)	1157 (28.7)		1941 (28.2)	1136 (28.2)	
Infection site, *n* (%)						
Blood	33 (0.5)	190 (4.7)	0.27	83 (1.2)	49 (1.2)	0.00
Genitourinary	200 (2.9)	255 (6.3)	0.16	304 (4.4)	178 (4.4)	0.00
Intra-abdominal	1504 (21.8)	981 (24.3)	0.06	1575 (22.9)	922 (22.9)	0.00
Pulmonary	884 (12.8)	660 (16.4)	0.10	1085 (15.8)	635 (15.8)	0.00
Others	60 (0.9)	69 (1.7)	0.07	89 (1.3)	52 (1.3)	0.00
Broad-spectrum initiation,^a^ *n* (%)	1516 (22.0)	1786 (44.3)	0.49	2319 (33.7)	1357 (33.7)	0.00
CCI (%)			0.08			0.00
0	3261 (47.4)	1750 (43.4)		3081 (44.7)	1803 (44.7)	
≥1	3625 (52.6)	2279 (56.6)		3805 (55.3)	2227 (55.3)	
Medical history,^b^ *n* (%)						
Chemotherapy	1323 (19.2)	1054 (26.2)	0.17	1620 (23.5)	948 (23.5)	0.00
Radiation	84 (1.2)	61 (1.5)	0.03	101 (1.5)	59 (1.5)	0.00
Surgery	1192 (17.3)	326 (8.1)	0.28	683 (9.9)	400 (9.9)	0.00
Immunosuppressive agents	1964 (28.5)	1414 (35.1)	0.14	2214 (32.2)	1296 (32.2)	0.00
After COVID-19 pandemic, *n* (%)	3066 (44.5)	2089 (51.8)	0.15	3352 (48.7)	1961 (48.7)	0.00
ICU admission, *n* (%)	240 (3.5)	274 (6.8)	0.15	325 (4.7)	190 (4.7)	0.00
Palliative care intervention, *n* (%)	145 (2.1)	51 (1.3)	0.07	104 (1.5)	61 (1.5)	0.00
Oxygen inhalation, *n* (%)	1635 (23.7)	1042 (25.9)	0.05	1741 (25.3)	1019 (25.3)	0.00
Mechanical ventilation, *n* (%)	78 (1.1)	51 (1.3)	0.01	84 (1.2)	49 (1.2)	0.00
Vasopressor use, *n* (%)	108 (1.6)	94 (2.3)	0.06	114 (1.7)	67 (1.7)	0.00
BT <36°C or >38°C, *n* (%)	1679 (24.4)	1907 (47.3)	0.49	2524 (36.6)	1477 (36.6)	0.00
GCS <15, *n* (%)	3653 (53.0)	2602 (64.6)	0.24	4096 (59.5)	2397 (59.5)	0.00
HR >90 beats/min, *n* (%)	305 (4.4)	305 (7.6)	0.13	410 (6.0)	240 (6.0)	0.00
sBP ≤100 mmHg, *n* (%)	1994 (29.0)	1418 (35.2)	0.13	2238 (32.5)	1309 (32.5)	0.00
Albumin, mean (SD), g/dL	3.0 (0.7)	2.9 (0.6)	0.21	2.9 (0.7)	2.9 (0.6)	0.00
ANC <1000 cells/µL, *n* (%)	200 (2.9)	188 (4.7)	0.09	250 (3.6)	147 (3.6)	0.00
Creatinine, mean (SD), mg/dL	1.1 (1.2)	1.0 (1.0)	0.01	1.0 (1.0)	1.0 (1.0)	0.00
CRP, mean (SD), mg/dL	8.5 (7.8)	11.3 (8.7)	0.34	10.3 (8.5)	10.3 (8.1)	0.00
Haemoglobin, mean (SD), g/dL	11.1 (2.4)	10.5 (2.4)	0.24	10.7 (2.4)	10.7 (2.4)	0.00
Plt, mean (SD), 10^4^/µL	26.8 (13.2)	24.9 (13.5)	0.14	26.0 (13.5)	26.0 (13.6)	0.00
T-Bil, mean (SD), mg/dL	2.1 (3.9)	1.6 (2.8)	0.15	1.7 (3.0)	1.7 (3.2)	0.00
WBC >12 000 cells/µL, *n* (%)	2264 (32.9)	1539 (38.2)	0.11	2550 (37.0)	1492 (37.0)	0.00
Number of hospital beds, *n* (%)			0.32			0.00
<300	464 (6.7)	124 (3.1)		277 (4.0)	162 (4.0)	
300–499	3983 (57.8)	1889 (46.9)		3570 (51.8)	2089 (51.8)	
≥500	2439 (35.4)	2016 (50.0)		3039 (44.1)	1778 (44.1)	
Rural hospital	697 (10.1)	346 (8.6)	0.05	631 (9.2)	369 (9.2)	0.00

ANC, absolute neutrophil count; BC, blood culture group; BT, body temperature; CCI, Charlson Comorbidity Index; COVID-19, coronavirus disease 2019; CRP, C-reactive protein; GCS, Glasgow Coma Scale; HR, heart rate; NC, no blood culture group; Plt, platelet count; sBP, systolic blood pressure; SMD, standardized mean difference; T-Bil, total bilirubin.

^a^Patients were initially started on broad-spectrum antibiotics.

^b^Patients underwent treatment within 4 weeks prior to antimicrobial initiation.

**Figure 3.**
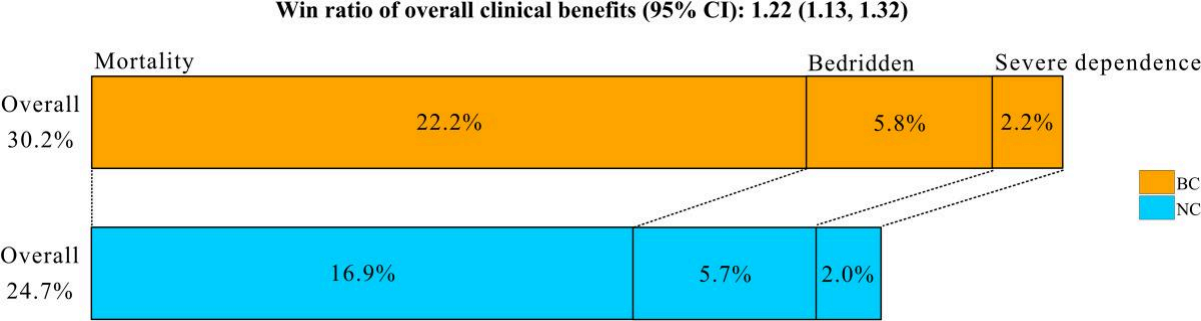
Adjusted win ratio of the composite primary outcome and its components using a non-parametric generalized pairwise comparison after overlap weighting. BC, blood culture group; NC, no blood culture group.

Using the weighted adjusted win ratio, the BC group showed more favourable outcomes than the NC group (30.2% versus 24.7%), with a win ratio of 1.22 (95% CI, 1.13–1.32; *P* < 0.001). The BC group showed higher win rates for mortality (22.2% versus 16.9%), with marginally higher win rates for functional status (bedridden: 5.8% versus 5.7%; severe dependence: 2.2% versus 2.0%) than the NC group (Figure 3). Subgroup analyses showed the win ratios were particularly higher in patients with prior chemotherapy or immunosuppressive agents, but no significant difference in win ratio between groups in those aged 18–64 years, with BMI ≥ 25.0 kg/m^2^, and with CCI = 0. Sensitivity analyses showed no significant difference in win ratio between groups only for patients admitted after the COVID-19 pandemic (Figure S3a). Although the BC group showed higher win rates for mortality than the NC group in all subgroups, the BC group tended to show lower win rates for functional status than the NC group, particularly in subgroups that showed no significant differences in the win ratio between BC and NC (Table S6). Although ties accounted for 45.1% of all comparisons, win ratios and win odds showed consistent patterns (Figure S3b).

The median LOS was 20.0 days (IQR: 11.0–34.0) in the BC group and 19.0 days (IQR: 10.0–33.0) in the NC group, with a difference of 1.0 days (95% CI, 0.0–1.9; *P* = 0.04) between groups. The BC group had longer DOTs per 100-LOS for all IV antimicrobials than the NC group [60.6 (95% CI, 59.7–61.5) versus 57.5 (56.8–58.5); *P* < 0.01], but DOTs per 100-LOS for broad-spectrum IV antimicrobials were similar between the groups [24.0 (95% CI, 23.0–25.0) versus 23.5 (95% CI, 22.4–24.7); *P* = 0.54]. The median total healthcare cost was 5851.0 USD (IQR: 3374.8–9960.5) in the BC group and 5569.4 USD (IQR: 3189.2–9983.0) in the NC group, with a difference of 345.0 USD (95% CI, 72.5–628.1; *P* = 0.01) between groups. The costs of medical consultation, medication, laboratory tests, hospital stay, and others were significantly higher in the BC group than in the NC group (Table 2).

**Table 2.** Comparison of secondary outcomes between the NC and BC groups after overlap weighting

Secondary outcomes	NC *n* = 6886	BC *n* = 4029	*P*
LOS, median [IQR]	19.0 [10.0, 33.0]	20.0 [11.0, 34.0]	<0.01
DOTs of all antimicrobials per 100-LOS,^a^ mean (95% CI)	57.5 (56.8, 58.5)	60.6 (59.7, 61.5)	<0.01
DOTs of broad-spectrum antimicrobials per 100-LOS,^b^ mean (95% CI)	23.5 (22.4, 24.7)	24.0 (23.0, 25.0)	0.54
Cost, median [IQR], USD			
Total	5569.4 [3189.2, 9983.0]	5851.0 [3374.8, 9960.5]	0.01
Medical consultation	76.7 [37.3, 141.2]	80.7 [42.0, 148.8]	<0.01
Medication	436.9 [196.2, 996.6]	467.4 [216.0, 1095.4]	<0.01
Surgical procedure	256.2 [22.9, 1892.0]	201.1 [14.1, 1462.1]	<0.01
Laboratory tests	581.0 [327.0, 1068.7]	666.5 [416.3, 1171.6]	<0.01
Hospital stay	3161.5 [1872.0, 5157.8]	3362.5 [2008.5, 5283.1]	<0.01
Others	0.0 [0.0, 136.7]	0.0 [0.0, 175.7]	<0.01

BC, blood culture group; DOTs, days of therapy; LOS, length of stay from antimicrobial initiation to discharge; NC, no blood culture group; USD, US dollars.

^a^DOTs of all IV antimicrobials per 100-LOS during hospitalization.

^b^DOTs of broad-spectrum IV antimicrobials per 100-LOS during hospitalization.

## Discussion

Blood cultures were associated with reduced in-hospital mortality in patients with stage IV solid cancer receiving antimicrobial therapy in unplanned admission. Sensitivity analyses confirmed the robustness of our findings. Subgroup analyses showed that mortality was reduced particularly in patients with prior chemotherapy or immunosuppressive agents. Blood cultures showed slight improvements in functional outcomes with additional costs and longer LOS.

Our findings complement recent emergency department guidelines recommending blood cultures for immunocompromised patients based on expert opinion.^6^ We provided insights into supporting this recommendation in patients with stage IV solid cancer, who were vulnerable to infections due to compromised immune systems.^2,3^ Blood cultures were associated with reduced mortality particularly in patients with prior chemotherapy or immunosuppressive agents, suggesting additional immunosuppression made patients more vulnerable to infections.

Previous studies^10,12^ focused primarily on mortality, whereas we also quantified functional outcomes. Blood cultures showed slight improvements in functional outcomes, suggesting that patients with stage IV solid cancer might require prolonged time for functional recovery, even with appropriate antimicrobial therapy. In subgroups with better general condition (aged 18–64, BMI ≥ 25.0 kg/m^2^, CCI = 0),^20,36,37^ the BC and NC groups had lower mortality rates than the overall population without significant differences in win ratio. This suggests that empirical therapy might be sufficient when the patient’s general condition is favourable. For patients admitted after the COVID-19 pandemic, the win ratio showed no significant difference between the BC and NC groups despite mortality rates similar to the overall population. This finding indicates that pandemic-related delays in cancer rehabilitation^38^ might lead to functional recovery impairments, which can offset blood culture benefits in composite outcomes.

Blood cultures were associated with longer LOS and higher total costs, as in a previous study.^9^ Although the detailed costs for blood cultures cannot be precisely isolated from our data, the additional laboratory costs may reflect the blood culture–related costs (approximately 42–64 USD per test in Japan), considering multiple tests may be performed during hospitalization. Additionally, the clinical challenge of potential contaminants should be considered. The detection of CoNS—a common contaminant that can also be a true pathogen in patients with immunosuppression^39^—in some positive cultures highlights the risk of unnecessary antibiotic treatment. Alongside these challenges, our analysis provided findings consistent with antimicrobial stewardship. Blood cultures were associated with extension of overall IV antimicrobial use but no significant extension of broad-spectrum IV antimicrobial use. In the BC group, patients with negative cultures had a shorter duration of overall antimicrobial therapy than those with positive cultures. Moreover, patients in whom susceptible bacteria were detected had a shorter duration of broad-spectrum therapy than those with resistant bacteria, consistent with de-escalation. These findings suggest that blood cultures contribute to antimicrobial stewardship by optimizing antimicrobial use. Given that antimicrobial resistance is a critical concern in patients with cancer,^40^ appropriate antimicrobial use is crucial. Thus, considering both the benefits and the need for resource allocation,^7,41^ our findings demonstrate the importance of careful patient selection, particularly in resource-limited settings.

These findings enable patients, families and healthcare providers to make informed decisions on blood culture based on the balance between mortality reduction and associated burdens.^9,10^ Healthcare providers could recommend blood cultures for most patients with stage IV solid cancer, particularly those receiving chemotherapy or immunosuppressive agents. However, a more careful decision may be required among patients aged 18–64 and ≥85 years, in whom the reduction associated with BC was more limited and was not statistically significant. For patients aged 18–64 years, who had lower mortality rates than the overall population, this finding may reflect better general condition and immune responses,^20^ suggesting the incremental benefit of BC over empirical therapy may be smaller in this less vulnerable population. Among patients aged ≥85 years, who had higher mortality rates than the overall population, this finding may reflect a ceiling to the benefit of aggressive therapy^8^ and highlights the importance of shared decision-making to align BC with patient preferences and goals of care. This focus on patient-centred goals, particularly in patients aged ≥85 years, connects to the role of palliative care and resource allocation. Palliative care interventions can reduce unnecessary antimicrobial use in terminal care settings.^4^ Given that 60% of Japanese patients with cancer died in general wards,^42^ increasing palliative care implementation could be beneficial. Healthcare providers should also offer active rehabilitation to support functional recovery. Furthermore, the mortality reduction with additional costs could help policymakers allocate resources to implement blood cultures according to the patient characteristics.

This study has some limitations. First, it was based on data from 63 Japanese hospitals, which might limit generalizability. However, blood culture implementation rates, culture positivity and bacterial profiles were similar to those in previous studies,^10,12,43^ suggesting comparable infection management approaches.

Second, although we used overlap propensity-score weighting to balance potential confounders for imputed data, missing values or unmeasured confounding factors could have influenced our results. These unmeasured factors include performance status, the goals-of-care discussions, the type of chemotherapy (cytotoxic, molecular targeted or immunotherapeutic agents), marital status,^44^ and physician instructions for life-sustaining treatment.^4^

Third, our findings are applicable to patients with stage IV cancer whose suspected infection was severe enough to start IV antimicrobials within 1 day of an unplanned admission. The risk–benefit balance may differ in less severe cases where it may be clinically appropriate to await initiation of antimicrobial therapy until blood culture results are available.

Fourth, our study may have been underpowered for the interaction tests owing to the smaller sample sizes in certain subgroups, such as patients aged ≥85 years. These findings should be interpreted with caution and require confirmation in larger studies.

Fifth, we could not assess long-term outcomes after discharge. Because older patients (aged ≥65 years) with cancer who become bedridden have poor survival (median 62 days),^20^ further studies with follow-up after discharge are needed to confirm the relationship between blood cultures and long-term outcomes.

Finally, our ability to assess whether antimicrobial selection was appropriate was limited. A comparison between groups was not possible because culture results were only available for the BC group. Even within the BC group, our analysis was constrained as the database lacked information on when results were reported or acted upon by healthcare providers. This data gap prevented us from establishing a causal link between culture results and subsequent clinical decisions, hindering a full assessment of whether antimicrobial selection was appropriate. Further research is needed to investigate how blood cultures guide antimicrobial selection in infection management in patients with stage IV cancer.

### Conclusion

In patients with stage IV cancer, blood cultures were associated with reduced mortality but additional LOS and healthcare costs. These findings suggests that blood cultures should be prioritized for most patients with stage IV cancer in unplanned admission, particularly those with immunosuppression.

## Supplementary Material

dkaf368_Supplementary_Data

## Data Availability

The datasets generated or analysed during this study are not publicly available due to the inclusion of sensitive personal information. The analytic code is available from the corresponding author on request.

## Supplementary data

Figures S1 to S3 and Tables S1 to S6 are available as Supplementary data at *JAC* Online.
