# Supplementary material for: Risk–benefit balance of blood cultures among patients with stage IV cancer in unplanned admission: a nationwide propensity score–weighted study in Japan
Source: J Antimicrob Chemother. 2025 Oct 3;80(12):3311–9. doi: 10.1093/jac/dkaf368 (PMC12670161; doi:10.1093/jac/dkaf368)
Supplement: dkaf368_Supplementary_Data [file dkaf368_supplementary_data.docx]

Table S1. ICD-10 codes of variables used as covariates

| Disease | ICD-10 codes |
| --- | --- |
| Primary cancer site |  |
| Breast | C50 |
| Colorectal | C18, C19, C20, C785 |
| Liver | C22, C787 |
| Lung | C34, C780 |
| Pancreas | C25 |
| Stomach | C16 |
| Others | C00, C01, C02, C03, C04, C05, C06, C07, C08, C09, C10, C11, C12, C13, C14, C15, C17, C21, C23, C24, C26, C30, C31, C32, C33, C37, C38, C39, C40, C41, C43, C44, C45, C46, C47, C48, C49, C51, C52, C53, C54, C55, C56, C57, C58, C60, C61, C62, C63, C64, C65, C66, C67, C68, C69, C70, C71, C72, C73, C74, C75, C76, C77, C781, C782, C783, C784, C786, C788, C79, C80, C96, C97 |
| Infectious site |  |
| Blood | A327, A391, A392, A393, A394, A399, A400, A401, A402, A403, A408, A409, A410, A411, A412, A413, A414, A415, A418, A419 |
| Genitourinary | N10, N111, N118, N119, N12, N136, N151, N159, N300, N302, N308, N309, N340, N341, N342, N361, N390, T835 |
| Intra-Abdominal | K352, K353, K358, K36, K37, K571, K572, K573, K579, K610, K611, K612, K613, K614, K630, K650, K658, K659, K750, K751, K800, K801, K803, K804, K810, K811, K819, K830, K831, K858, K859 |
| Pulmonary | J13, J14, J150, J151, J152, J153, J154, J155, J156, J157, J158, J159, J160, J180, J181, J182, J188, J189, J690, J850, J851, J852, J853, J958 |
| Others | L00, L010, L011, L020, L021, L022, L023, L024, L028, L029, L030, L031, L032, L033, L038, L039, L040, L041, L042, L043, L049, L059, L080, L081, L089, L88, M0001, M0002, M0005, M0006, M0009, M0019, M0025, M0029, M0089, M0091, M0092, M0093, M0094, M0095, M0096, M0097, M0099, M4621, M4622, M4624, M4626, M4628, M4629, M4632, M4633, M4634, M4635, M4636, M4637, M4639, M4652, M4654, M4656, M4659, M6009, M7260, M7263, M7265, M7266, M7267, M7269, M7289, M8605, M8606, M8609, M8616, M8619, M8629, M8630, M8655, M8656, M8659, M8662, M8665, M8666, M8669, M8689, M8691, M8692, M8693, M8694, M8695, M8696, M8697, M8698, M8699 |

Table S2. Missing variables of patients with stage IV cancer

| Missing variable, n (%) | **NC**  ***n* = 6886** | **BC**  ***n* = 4029** | **SMD** |
| --- | --- | --- | --- |
| BMI | 274 (4.0) | 143 (3.5) | 0.02 |
| Functional status at admission | 673 (9.8) | 432 (10.7) | 0.03 |
| BT | 158 (2.3) | 106 (2.6) | 0.02 |
| HR | 635 (9.2) | 461 (11.4) | 0.07 |
| sBP | 178 (2.6) | 103 (2.6) | 0.00 |
| Albumin | 667 (9.7) | 336 (8.3) | 0.05 |
| ANC | 1071 (15.6) | 686 (17.0) | 0.04 |
| Creatinine | 385 (5.6) | 93 (2.3) | 0.17 |
| CRP | 367 (5.3) | 82 (2.0) | 0.18 |
| Haemoglobin | 253 (3.7) | 68 (1.7) | 0.12 |
| Plt | 253 (3.7) | 68 (1.7) | 0.12 |
| T-Bil | 425 (6.2) | 137 (3.4) | 0.13 |
| WBC | 423 (6.1) | 125 (3.1) | 0.15 |

ANC = absolute neutrophil count; BC = blood culture group; BT = body temperature; CRP = C-reactive protein; HR = heart rate; NC = no blood culture group; Plt = platelet count; sBP = systolic blood pressure; SMD = standardized mean difference; T-Bil = total bilirubin; WBC = white blood cell count.

Table S3. Blood culture results in patients with stage IV cancer who underwent blood culture

| Blood-culture results | n (%)  *n* = 4029 |
| --- | --- |
| Negative | 3366 (83.5) |
| Positive | 663 (16.5) |
| Type of bacteria |  |
| *Acinetobacter* spp. | 4 (0.1) |
| Enterobacteriaceae | 356 (8.8) |
| *Escherichia coli* | 161 (4.0) |
| *Klebsiella pneumoniae* | 106 (2.6) |
| *Pseudomonas aeruginosa* | 16 (0.4) |
| *Staphylococcus aureus* | 46 (1.1) |
| Coagulase-negative staphylococci | 84 (2.1) |
| Antimicrobial susceptibility |  |
| Susceptible | 222 (5.5) |
| Resistant | 441 (10.9) |
| Type of antimicrobial resistant bacteria |  |
| Carbapenem-resistant Enterobacteriaceae | 2 (0.0) |
| Fluoroquinolone-resistant *Escherichia coli* | 39 (1.0) |
| Methicillin-resistant *Staphylococcus aureus* | 19 (0.5) |
| Third-generation cephalosporin-resistant *Escherichia coli* | 24 (0.6) |
| Third-generation cephalosporin-resistant *Klebsiella pneumoniae* | 7 (0.2) |

Table S4. DOTs of antimicrobials based on bacteria detection in patients with stage IV cancer who underwent blood culture

| Variable, mean (95%CI) | Negative  *n* = 3366 | Positive  *n* = 663 | *P* |
| --- | --- | --- | --- |
| DOTs of all antimicrobials per 100-LOS^a^ | 61.4 (60.4, 62.4) | 69.5 (67.3, 71.8) | <.001 |
| DOTs of broad-spectrum antimicrobials per 100-LOS^b^ | 29.5 (28.3, 30.7) | 31.0 (28.4, 33.6) | .31 |

a: “DOTs per 100-LOS for overall antimicrobials” means DOTs of all intravenous antimicrobials per 100-LOS during hospitalisation.

b: “DOTs per 100-LOS for broad-spectrum antimicrobials” means DOTs of broad-spectrum intravenous antimicrobials per 100-LOS

during hospitalisation.

DOTs =days of therapy; LOS = length of stay from antimicrobial initiation to discharge.

Table S5. DOTs of antimicrobials based on the antimicrobial susceptibility in patients with stage IV cancer who underwent blood culture

| Variable, mean (95%CI) | Susceptible  *n* = 222 | Resistant  *n* = 441 | *P* |
| --- | --- | --- | --- |
| DOTs of all antimicrobials per 100-LOS^a^ | 67.4 (63.6, 71.2) | 70.6 (67.8, 73.4) | .18 |
| DOTs of broad-spectrum antimicrobials per 100-LOS^b^ | 27.2 (23.1, 31.4) | 32.8 (29.6, 36.1) | .04 |

a: “DOTs per 100-LOS for overall antimicrobials” means DOTs of all intravenous antimicrobials per 100-LOS during hospitalisation.

b: “DOTs per 100-LOS for broad-spectrum antimicrobials” means DOTs of broad-spectrum intravenous antimicrobials per 100-LOS

during hospitalisation.

DOTs =days of therapy; LOS = length of stay from antimicrobial initiation to discharge.

Table S6. Win ratio for each primary outcome in patients with and without blood culture after overlap weighting

|  | Overall | | Mortality | | Bedridden | | Severe dependence | |
| --- | --- | --- | --- | --- | --- | --- | --- | --- |
| Category | NC | BC | NC | BC | NC | BC | NC | BC |
| All (%) | 24.7 | 30.2 | 16.9 | 22.2 | 5.7 | 5.8 | 2.0 | 2.2 |
| Subgroup analysis (%) |  |  |  |  |  |  |  |  |
| Age, years |  |  |  |  |  |  |  |  |
| 18–64 | 18.5 | 22.5 | 15.2 | 17.6 | 2.5 | 3.3 | 0.8 | 1.6 |
| 65–74 | 23.7 | 27.5 | 16.5 | 21.4 | 5.0 | 4.1 | 2.1 | 1.9 |
| 75–84 | 25.1 | 32.8 | 16.6 | 24.1 | 6.2 | 6.5 | 2.3 | 2.2 |
| ≥85 | 30.5 | 37.1 | 20.3 | 25.1 | 8.2 | 9.3 | 1.9 | 2.8 |
| Sex |  |  |  |  |  |  |  |  |
| Male | 24.3 | 30.3 | 17.6 | 22.8 | 5.0 | 5.2 | 1.7 | 2.3 |
| Female | 25.3 | 30.0 | 15.7 | 21.2 | 7.1 | 6.8 | 2.5 | 2.0 |
| BMI, kg/m^2^ |  |  |  |  |  |  |  |  |
| <18.5 | 27.1 | 34.1 | 19.2 | 24.1 | 6.0 | 7.5 | 1.9 | 2.5 |
| 18.5–24.9 | 24.2 | 29.1 | 16.2 | 21.7 | 5.8 | 5.3 | 2.1 | 2.2 |
| ≥25.0 | 21.7 | 26.8 | 15.7 | 20.9 | 4.4 | 4.5 | 1.7 | 1.4 |
| Functional status |  |  |  |  |  |  |  |  |
| Not bedridden | 19.9 | 24.3 | 13.7 | 17.8 | 3.1 | 3.1 | 3.2 | 3.4 |
| Bedridden | 26.8 | 36.8 | 20.8 | 29.6 | 6.0 | 7.2 | 0.0 | 0.0 |
| CCI |  |  |  |  |  |  |  |  |
| 0 | 25.8 | 28.2 | 16.9 | 20.7 | 6.7 | 5.5 | 2.2 | 1.9 |
| ≥1 | 23.8 | 31.9 | 16.9 | 23.4 | 5.0 | 6.0 | 1.8 | 2.4 |
| Broad-spectrum initiation^a^ | 23.5 | 31.9 | 16.9 | 24.5 | 5.1 | 5.6 | 1.6 | 1.9 |
| Yes | 25.3 | 29.3 | 17.0 | 21.1 | 6.1 | 5.9 | 2.2 | 2.3 |
| No |  |  |  |  |  |  |  |  |
| Chemotherapy | 17.5 | 26.9 | 11.4 | 20.7 | 4.1 | 4.0 | 2.1 | 2.2 |
| Yes | 26.6 | 30.9 | 18.5 | 22.5 | 6.1 | 6.2 | 2.0 | 2.2 |
| No |  |  |  |  |  |  |  |  |
| Immunosuppressive agents | 19.8 | 30.1 | 15.3 | 23.6 | 3.0 | 4.3 | 1.4 | 2.3 |
| Yes | 27.0 | 30.1 | 17.7 | 21.6 | 7.0 | 6.4 | 2.3 | 2.1 |
| No |  |  |  |  |  |  |  |  |
| Sensitivity analysis (%) | 26.1 | 28.8 | 17.4 | 20.6 | 6.5 | 6.0 | 2.3 | 2.3 |
| After COVID-19 pandemic | 23.1 | 29.5 | 14.8 | 21.0 | 6.3 | 6.4 | 1.9 | 2.0 |
| Complete cases | 24.4 | 30.1 | 16.5 | 21.7 | 5.8 | 6.1 | 2.1 | 2.3 |
| Continuous use ≥3 days^b^ | 25.1 | 29.4 | 17.2 | 21.5 | 5.9 | 5.6 | 2.1 | 2.3 |
| Exclusion of BC after initiation^c^ | 23.3 | 29.0 | 16.5 | 21.8 | 4.7 | 5.0 | 2.1 | 2.2 |
| LOS ≤28 days | 23.5 | 31.9 | 16.9 | 24.5 | 5.1 | 5.6 | 1.6 | 1.9 |

a: “Broad-spectrum initiation” means that the patients initially used broad-spectrum antimicrobials.

b: “Continuous use ≥3 days” means that patients started intravenous antimicrobials and continued for at least 3 consecutive days.

c: “Exclusion of BC after initiation” means that we excluded patients who underwent blood culture after intravenous antimicrobials initiation from NC.

BC = blood culture group; CCI = Charlson Comorbidity Index; COVID-19 = coronavirus disease 2019; LOS = length of stay from antimicrobial initiation to discharge; NC = no blood culture group.


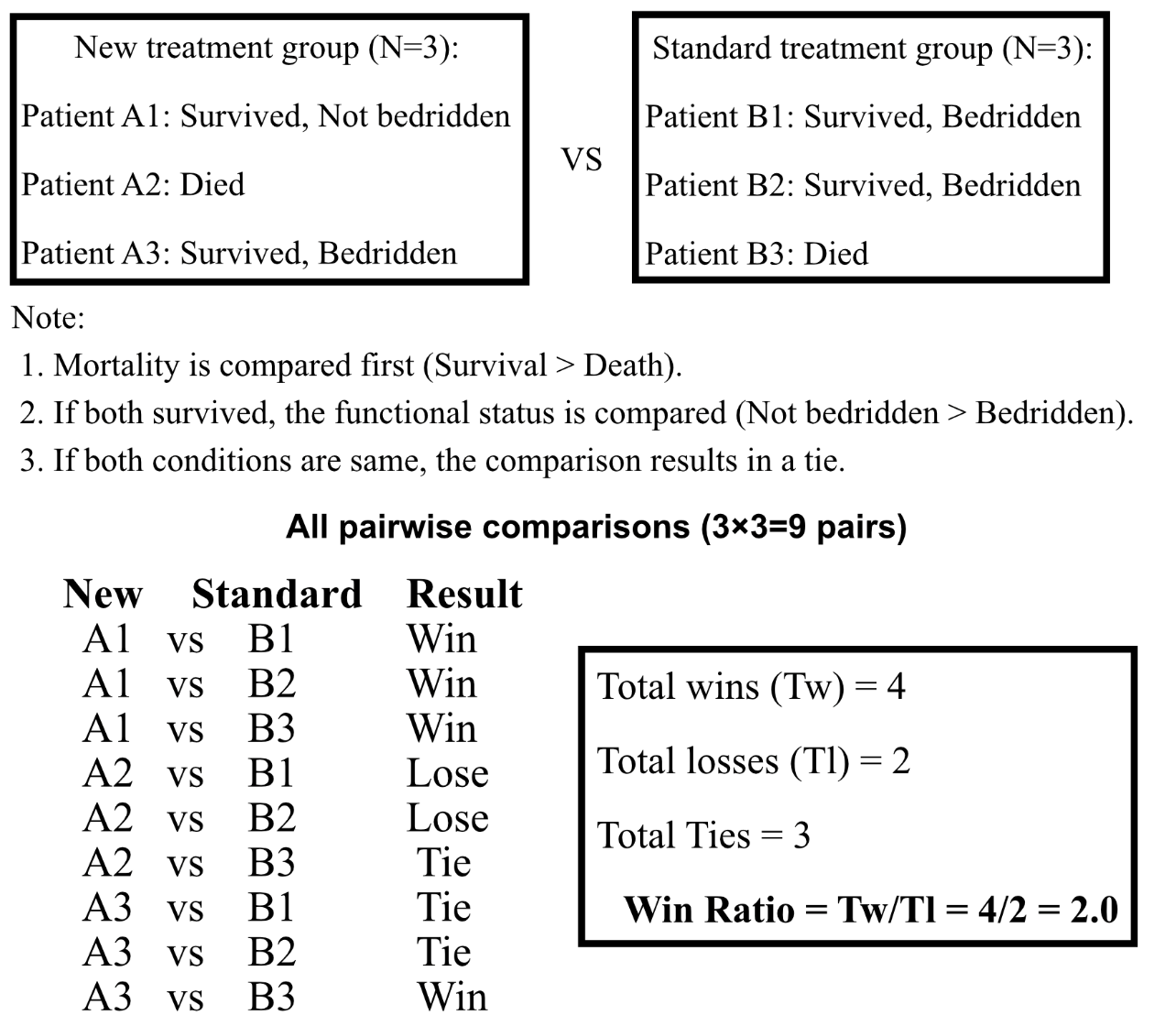


Figure S1. Schematic diagram of the win ratio approaches for composite endpoints (death and functional status).

This is a hypothetical example of a study that would have included 3 patients in the New treatment group and 3 patients in the Standard treatment group.


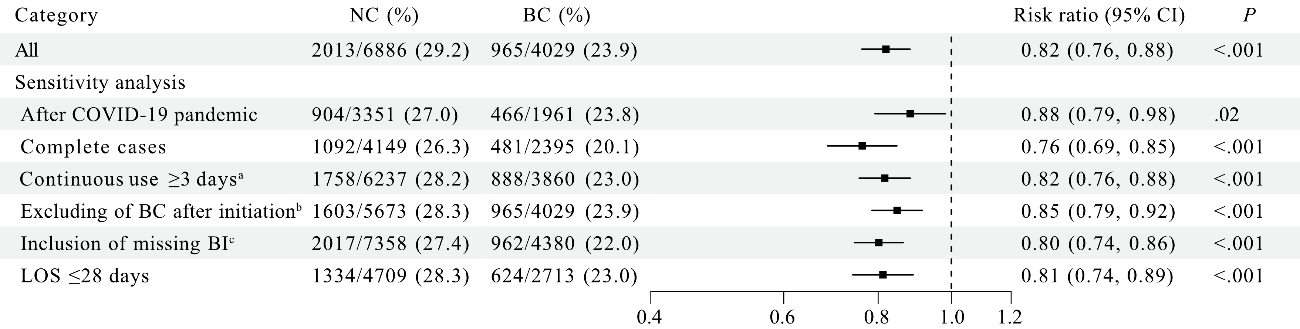
Figure S2. Forest plot showing the risk ratios by sensitivity analyses after overlap weighting.

a: “Continuous use ≥3 days” means that patients started intravenous antimicrobials and continued for at least 3 consecutive days.

b: “Exclusion of BC after initiation” means that we excluded patients who underwent blood culture after intravenous antimicrobials initiation from NC.

c: “Inclusion of missing BI” means that we added patients who had missing Barthel Index data at discharge.

BC = blood culture group; LOS = length of stay from antimicrobial initiation to discharge; NC = no blood culture group.


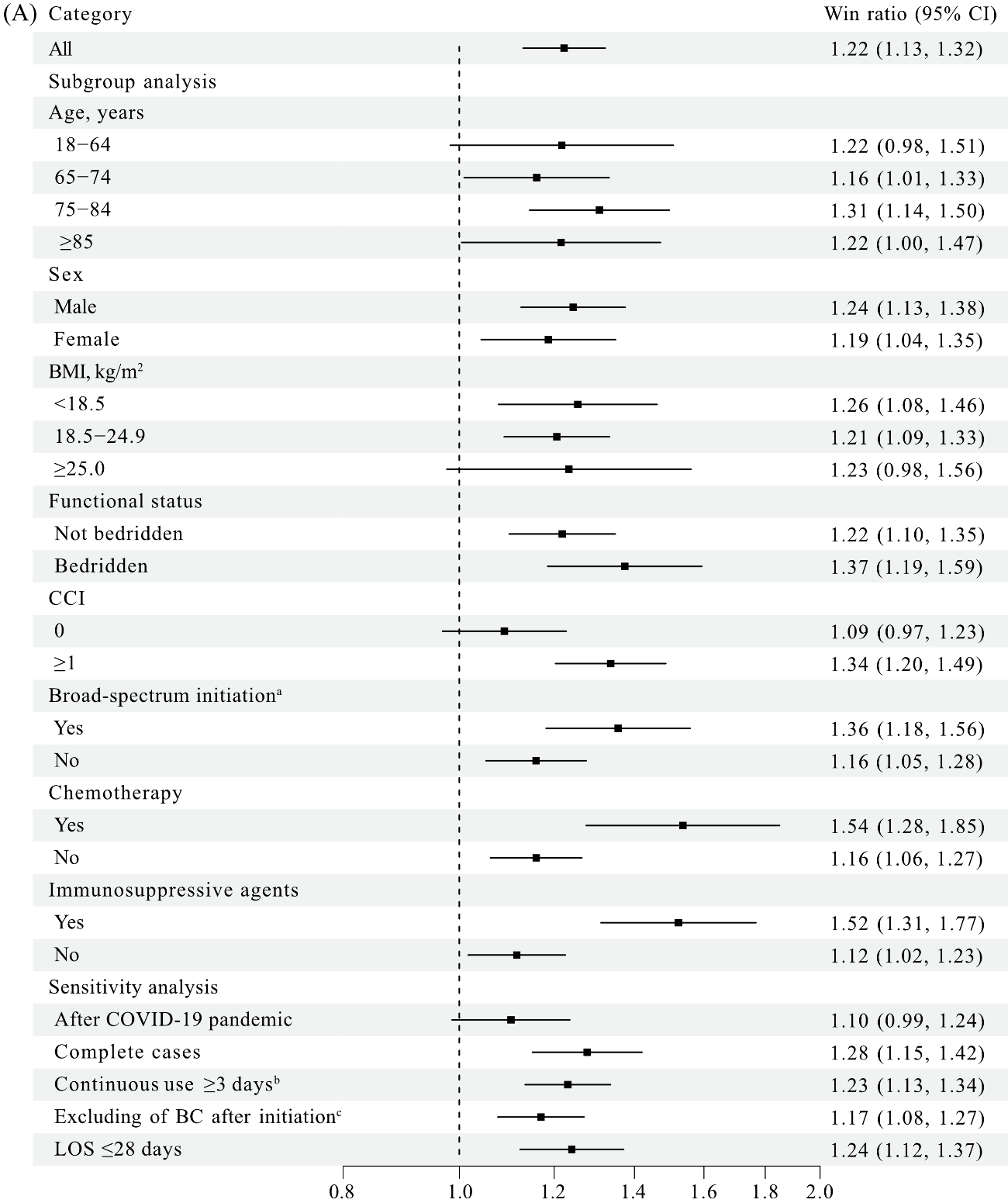


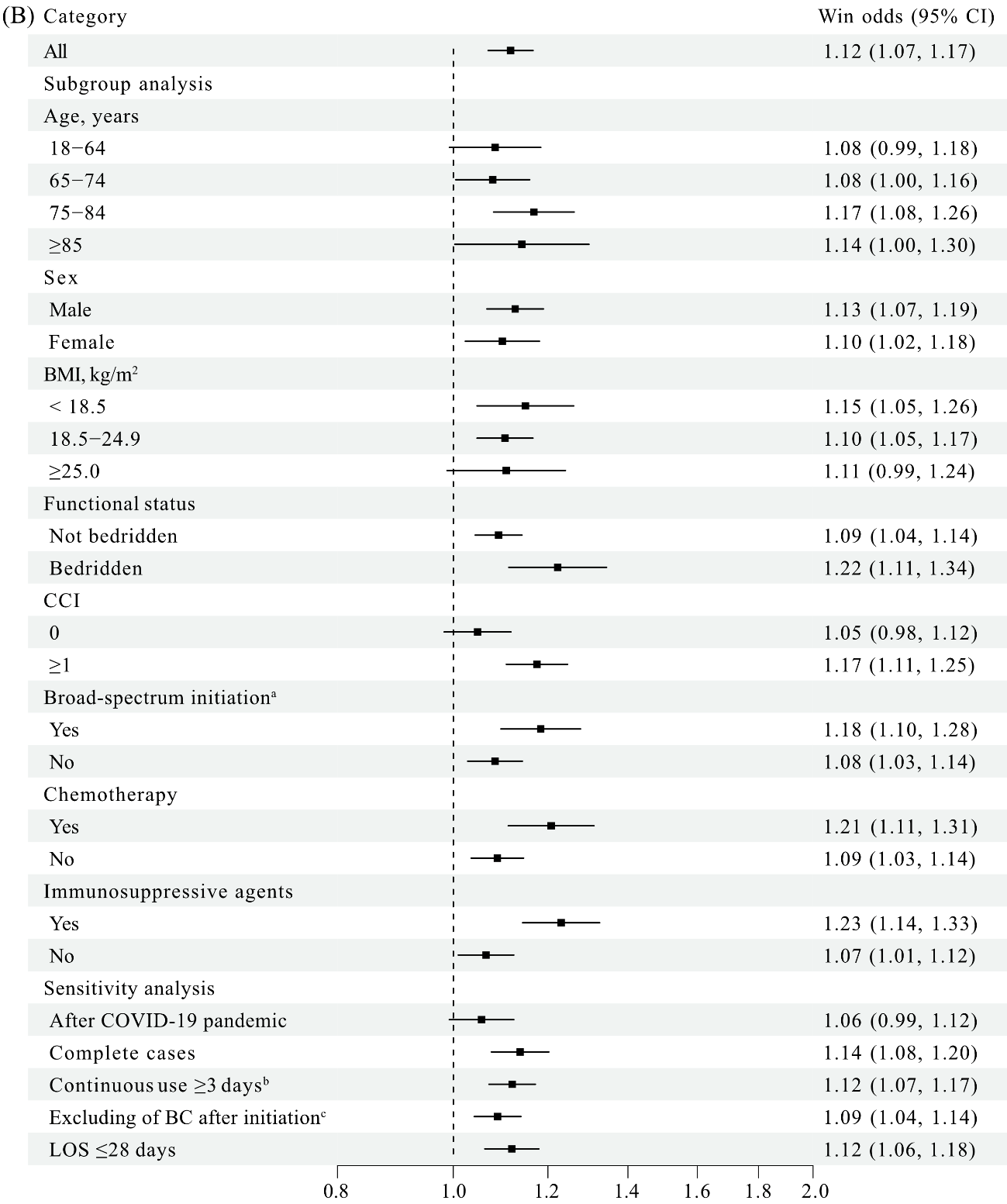


Figure S3. Forest plot showing the win-ratio approaches by subgroup and sensitivity analyses after overlap weighting.

(A) Forest plot showing the win ratios.

(B) Forest plot showing the win odds.

a: “Broad-spectrum initiation” means that the patients were initially started on broad-spectrum antibiotics.

b: “Continuous use ≥3 days” means that patients started intravenous antimicrobials and continued for at least 3 consecutive days.

c: “Exclusion of BC after initiation” means that we excluded patients who underwent blood culture after intravenous antimicrobials initiation from NC.

BC = blood culture group; CCI = Charlson Comorbidity Index; COVID-19 = coronavirus disease 2019; LOS = length of stay from antimicrobial initiation to discharge; NC = no blood culture group.
